# Disulfidptosis-related gene in acute myocardial infarction and immune microenvironment analysis: A bioinformatics analysis and validation

**DOI:** 10.1371/journal.pone.0314935

**Published:** 2024-12-12

**Authors:** Nan Huang, Chan Liu, Zheng Liu, Haibo Lei

**Affiliations:** 1 Clinical Pharmacy, Xiangtan Center Hospital, Xiangtan, Hunan Province, PR China; 2 Clinical Pharmacy, Liuyang People’s Hospital, Liuyang, Hunan Province, China; Townsville University Hospital, AUSTRALIA

## Abstract

Disulfidptosis is a newly discovered method of cell death. However, no studies have fully elucidated the role of disulfidptosis-related genes (DSRGs) in acute myocardial infarction (AMI). The potential role of DSRGs in AMI was analyzed through a comprehensive bioinformatics approach. Finally, hub genes were verified in vitro by qPCR. Sixteen DE-DSRGs were in the AMI. Thereafter, seven hub genes were determined by machine learning algorithms, which had potential diagnostic value in AMI. The risk model showed a robust diagnostic value (area under curve, AUC = 0.940). Prognostic analysis revealed the potential prognostic value of INF2 and CD2AP. Immune landscape analysis showed that hub genes were closely related to the immune microenvironment. By predictive analysis, we obtained four miRNAs, thirteen small molecule drugs, and five TFs closely related to hub genes. Experimental verification revealed that Slc3a2 and Inf2 were significantly up-regulated and Dstn was significantly down-regulated in the hypoxic model. Our study demonstrated that DSRGs are disorderedly expressed in AMI and identified seven hub genes through machine learning. In addition, a diagnostic model was constructed based on hub genes, providing a new perspective for the early diagnosis of AMI.

## 1. Introduction

AMI is a severe and potentially fatal condition that occurs when there is a blockage in blood flow to the heart muscle [1]. Common symptoms include chest pain or discomfort, shortness of breath, nausea, dizziness, and sweating. Treatments include drugs to dissolve clots and restore blood flow and procedures to open blocked arteries, such as angioplasty or stenting [2].

Previous studies have shown that AMI is a complex syndrome of multifactorial diseases. Risk factors include early family history, smoking, hypertension, dyslipidemia, and diabetes [1, 2]. When the coronary endothelial are damaged, inflammatory cells gather or accumulate fat and cholesterol, and other substances, leading to artery narrowing and plaque formation [3, 4]. When plaque ruptures, or after platelets gather on the plaque, blood clots form and block coronary blood flow, leading to ischemia and hypoxia damage to the heart muscle. The mechanism of myocardial injury is complex, involving inflammatory response, calcium overload, oxidative stress, mitochondrial membrane dysfunction, myocardial cell apoptosis, and autophagy [5, 6]. Although existing therapies have significantly reduced mortality and improved prognosis, AMI remains one of the leading causes of death worldwide [7]. Therefore, the development of a valuable early diagnosis model and clinical subtype classification model is of great significance for the diagnosis, prognosis, and treatment of AMI.

Disulfidptosis is a mode of cell death caused by disulfide stress [8]. Disulfide stress is an oxidation-reduction imbalance caused by intracellular NADPH depletion induced by glucose starvation. It mainly destroys the actin cytoskeleton, a network of protein fibers that provides mechanical support to cells and plays a key role in cell movement and division, thereby leading to cell death [9, 10]. In this study, bioinformatics methods were used to analyze the diagnostic, prognostic, and clinical subtype classification value of DSRGs in AMI and to explore the correlation between DSRGs and immune invasion.

## 2. Materials and methods

### 2.1 Data preprocess

GSE59867 [11], GSE62646 [12], GSE21545 [13], and GSE184073 [14] were obtained from the GEO database (Table 1). GSE59867 was utilized to analyze expression pattern and correlation of DSRGs, and to construct a risk model as a training set. GSE62646 was used to verify hub genes expression and risk model performance as a validation set. GSE21545 was utilized to evaluate the ability of hub genes to predict ischemic events via the K-M method using “tinyarray” packaged in R software. GSE184073 was employed to analyze the expression and differences of hub genes at the single-cell level. DSRGs and immune function-related gene set were obtained from previous literature.

**Table 1:** Data sets implemented for analysis.

Data set	Platform	Case samples	Control samples	Species
GSE59867	GPL6244	111	46	Homo sapien
GSE62646	GPL6244	14	28	Homo sapien
GSE21545	GPL570	76	21	Homo sapien
GSE184073	GPL24676	1	1	Homo sapien

### 2.2 Protein-protein interaction (PPI) and correlation analysis

DSRGs PPI was explored through the STRING website [15]. Correlation analysis in the text was performed via the “corrplot” package in R software.

### 2.3 Functional enrichment analysis

The "clusterProfiler 4.0" [16] package was utilized to perform Gene Ontology (GO) and Kyoto Encyclopedia of Genes and Genomes (KEGG) functional enrichment analysis of DSRGs and Cluster 1vs Cluster 2 differential genes. The results were visualized with histograms and bubble plots.

### 2.4 Prognostic and diagnostic values

Three machine learning algorithms, least absolute shrinkage and selection operator (LASSO), extreme gradient boosting (XGBoost), and support vector machine-recursive feature elimination (SVM-RFE), were used to screen for hub genes, and the first ten significant factors of the three algorithms were taken as the intersection of hub genes. Immediately afterward, risk scores were constructed using multi-factor logistic regression, and ROC curves were used to assess the diagnostic efficacy of hub genes and risk scores. Finally, Nomogram analysis was constructed for clinical application based on hub genes.

### 2.5 The construction of ceRNA, TFs prediction, and small molecule compounds screening

NetworkAnalyst (https://www.network.com/.ca/) [17] was used to construct the ceRNA network, TFs regulatory network, and small molecule compounds network of hub genes. These results were visualized by Cytoscape (version 3.8.0) [18].

### 2.6 Hub genes expression at the single-cell level

The GSE184073 dataset was employed to display the expression distribution of hub genes at the single-cell level. Brief steps: (1) Seurat object was builded by the "Seurat" package. (2) Quality control (QC) was conducted by removing low-quality cells. (3) Use the "FindVariableFeatures" function to select the top 2500 highly variable RNA features. (4) Perform principal component analysis (PCA) and t-distributed stochastic neighbor embedding (tSNE) based on the top 2500 highly variable genes to reduce dimensionality, cluster, and visualize. (5) Use the R package "SingleR" for automatic annotation of single-cell RNA seq data. (6) A matrix of hub genes expression was extracted and the expression landscape was displayed in a variety of cells via the “MySeuratWrappers” package. (7) Hub genes differential landscape was analyzed using the "ggplot2" R language package.

### 2.7 Consistency cluster analysis

Consensus clustering is a method for providing quantitative evidence for identifying potential cluster members in a dataset. This method has been widely used in cancer genomics, where new disease molecular subtypes have been discovered. In our study, consistency clustering analysis was performed on AMI patients based on hub gene expression levels using the “ConsensusClusterPlus” package in the GSE59867 dataset.

### 2.8 Immune landscape analysis

CIBERSORT is a method for characterizing cell composition in complex tissues based on gene expression data. Immune cell abundance was calculated by the “CIBERSORT” package in the GSE59867 dataset. ssGSEA was performed by the GSVA software package to predict the immune function score of each sample. The patterns of immune infiltration and immune function were compared between AMI and stable coronary artery disease (stable_CAD) groups by the “ggplot2” package. The correlation of hub genes with immune cells or immune function was explored via the “corrplot” package.

### 2.9 Differential expression analysis

The genetic difference analysis between the two groups was performed using the "limma" package, and the screening criteria for differential genes were that the absolute value of logFC was equal to 0.2 and the p-value was less than 0.05.

### 2.10 Weighted correlation network analysis (WGCNA) network construction and module identification

WGCNA [19] is a powerful bioinformatics analysis technique that allows for the identification of gene association patterns across different samples. By clustering genes with similar expression patterns and analyzing the relationship between modules and specific traits or phenotypes, WGCNA can provide valuable insights into gene function and regulation. In this study, we applied WGCNA to evaluate the training set by analyzing the first 40% variance of the gene matrix. Topology Overlap Matrix (TOM) was used for WGCNA network construction and module detection. Pearson correlation was applied to analyze the correlation of each module with the regulatory features of hub genes.

### 2.11 Cell culture and treatment

H9C2 cells (ProCell, Wuhan, China) were cultured in DMEM high-glucose medium supplemented with 10% fetal bovine serum and 1% penicillin/streptomycin double antibody. The culture condition was 37°C, 5% CO_2_. In order to construct the model of hypoxia and glucose deprivation, the cells of the model group were given glucose-free media without fetal bovine serum and double antibodies, and then placed in a three-gas incubator at 1% O_2_, 5% CO_2_, 94% N_2_, and kept at 37°C for 6 hours. Control cells were treated under normal conditions for 6 hours.

### 2.12 Hub genes expression was verified in vitro

The samples were homogenized with Trizol reagent (Simgene, Hangzhou), and total RNA was extracted according to the kit instructions. Total RNA was transcribed by HiScript® Q RT SuperMix for qPCR kit (Novozan, Nanjing) according to the instructions. The products obtained by reverse transcription were analyzed by qRT-PCR using ChamQ Universal SYBR qPCR Master Mix (Norweizan, Nanjing). The instrument used for qRT-PCR was MA-6000 Real-Time Quantitative Thermal Cycler (Shengxiang Biology, Changsha). The mRNA content of hub genes was determined with GAPDH as the internal reference. The primer sequence is as follows (Table 2).

**Table 2:** Primers for qPCR.

Gene	Primer sequence (5’-3’)
Gapdh-F	AGACAGCCGCATCTTCTTGT
Gapdh-R	TGATGGCAACAATGTCCACT
Slc7a11-F	AACCCAAGTGGTTCAGACGA
Slc7a11-R	ATCTCAGTCCTGGGCAGATG
Slc3a2-F	CTGGGACCAGAATGAGCGTT
Slc3a2-R	TTATGCCAGCAGGGAGGTTG
Rpn1-F	CCCGGGTCATTGAGGTTTCT
Rpn1-R	GCCTCTGGTAATCGTAGCGG
Nubpl-F	TTGGTGCTGATGGTGCAAGAA
Nubpl-R	GCTTTGGCCTCATCACTTTCA
Inf2-F	CCCTGGACAAGGCCCATAAG
Inf2-R	CGGCCTTCCACTTTAGCTGT
Cd2ap-F	TGGACCCAGTGGACCCTAAT
Cd2ap-R	TGCAATCCCAGCACAGTAAGA
Dstn-F	TTCGGAAATGCTCCACACCT
Dstn-R	GGTAACGCCGACATCTCCAA

### 2.13 Statistical analysis

Statistical analysis was performed using R software (version 4.1.2). The difference of continuous variables between the two groups was analyzed by Wilcoxon rank sum test. Log-rank test was used for comparing prognostic data. P<0.05 was considered statistically significant (*p-value< 0.05, **p-value< 0.01, ***p-value< 0.001, ****p-value< 0.0001).

## 3. Results

### 3.1 Identification of DE‐DSRGs in GSE59867

**Fig 1:**
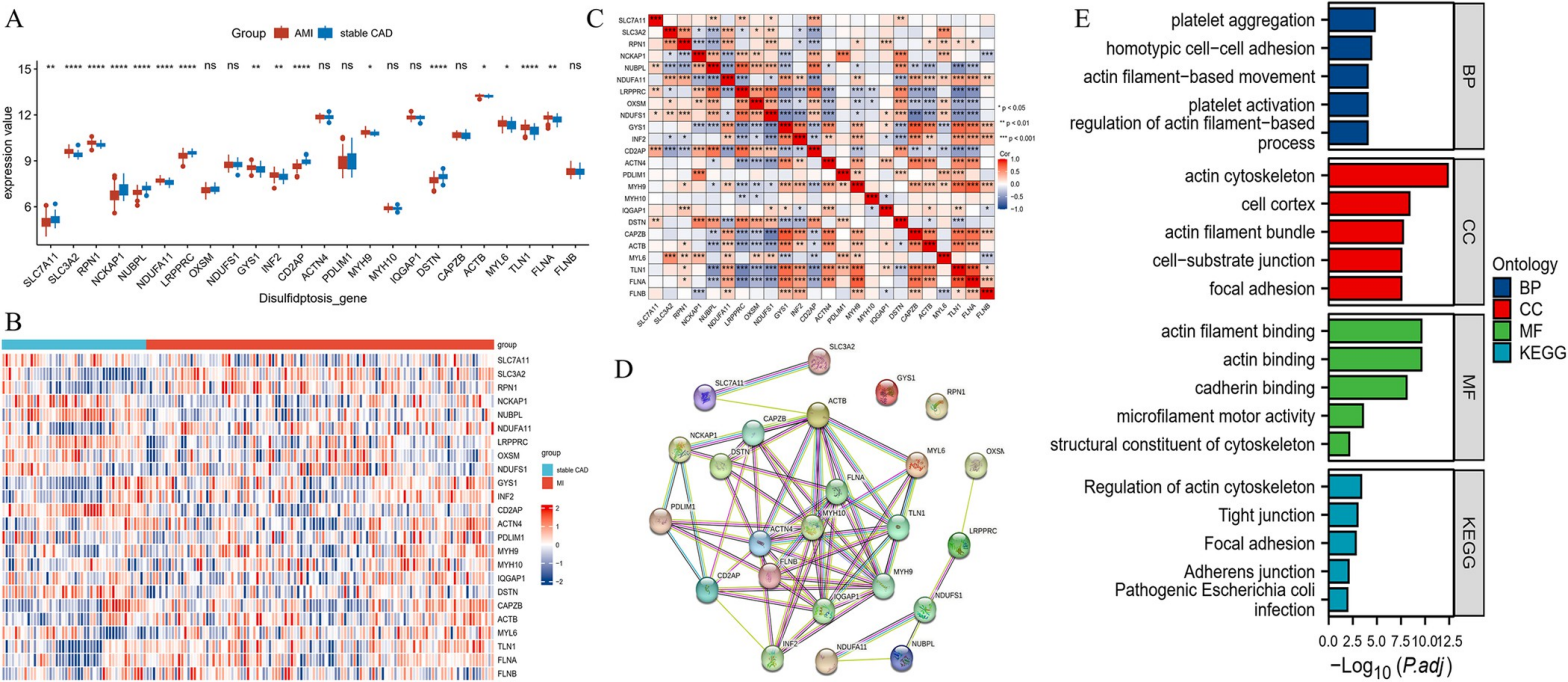
Differential expression of DSRGs, PPI, and functional enrichment analysis for DSRGs in AMI. A. Box plots show the expression patterns of DSRGs in AMI. B. Heatmap shows the expression patterns of DSRGs. C. Correlation heatmap of DSRGs. D. PPI network interworking diagram for DSRGs. E. Top5 functional enrichment histogram of DSRGs.

To explore the expression pattern of DSRGs in AMI, we analyzed the expression of DSRGs in GSE59867. We found that most DSRGs were disorderedly expressed in AMI, where SLC3A2, RPN1, NDUFA11, GYS1, INF2, MYH9, ACTB, MYL6, TLN1, FLNA were significantly upregulated, while SLC7A11, NCKAP1, NUBPL, LRPPRC, CD2AP, DSTN were significantly downregulated in AMI (Fig 1A). The heatmap further demonstrates the expression of DSRGs in different samples (Fig 1B). In addition, correlation analysis showed that there were complex correlations among DSRGs. Among them, TLN1 had the largest positive correlation with FLNA (r = 0.774, p = 0.000), and NDUFS1 had the largest negative correlation with CAPZB (r = -0.613, p = 0.000) (Fig 1C; S1, S2 Tables).

### 3.2 PPI, GO, KEGG analysis of DSRGs

PPI network construction is important for understanding the functional relationship between proteins. In our study, the DSRGs PPI network was constructed through the STRING website. The results showed that most DSRGs proteins, except GYS1 and RPN1, had close interactions with each other (Fig 1D). Furthermore, in order to reveal the functionality of DSRGs, GO, KEGG analysis was performed by the "clusterProfiler 4.0" package. Fig 1E showed that significantly enriched biological process (BP) included platelet aggregation, homotypic cell−cell adhesion, actin filament−based movement, platelet activation and regulation of actin filament−based process; platelet activation, and regulation of actin filament−based process; significantly enriched cellular component (CC) included actin cytoskeleton, cell cortex, actin filament bundle, cell−substrate junction, focal adhesion; obviously enriched molecular functions (MF) included actin filament binding, actin binding, cadherin binding, microfilament motor activity, structural constituent of cytoskeleton; Structural constituent of cytoskeleton; significantly enriched KEGG signaling pathways included Regulation of actin cytoskeleton, Tight junction, Focal adhesion, Adherens junction, Pathogenic Escherichia coli infection (S3 Table).

### 3.3 Diagnostic model construction by machine learning

**Fig 2:**
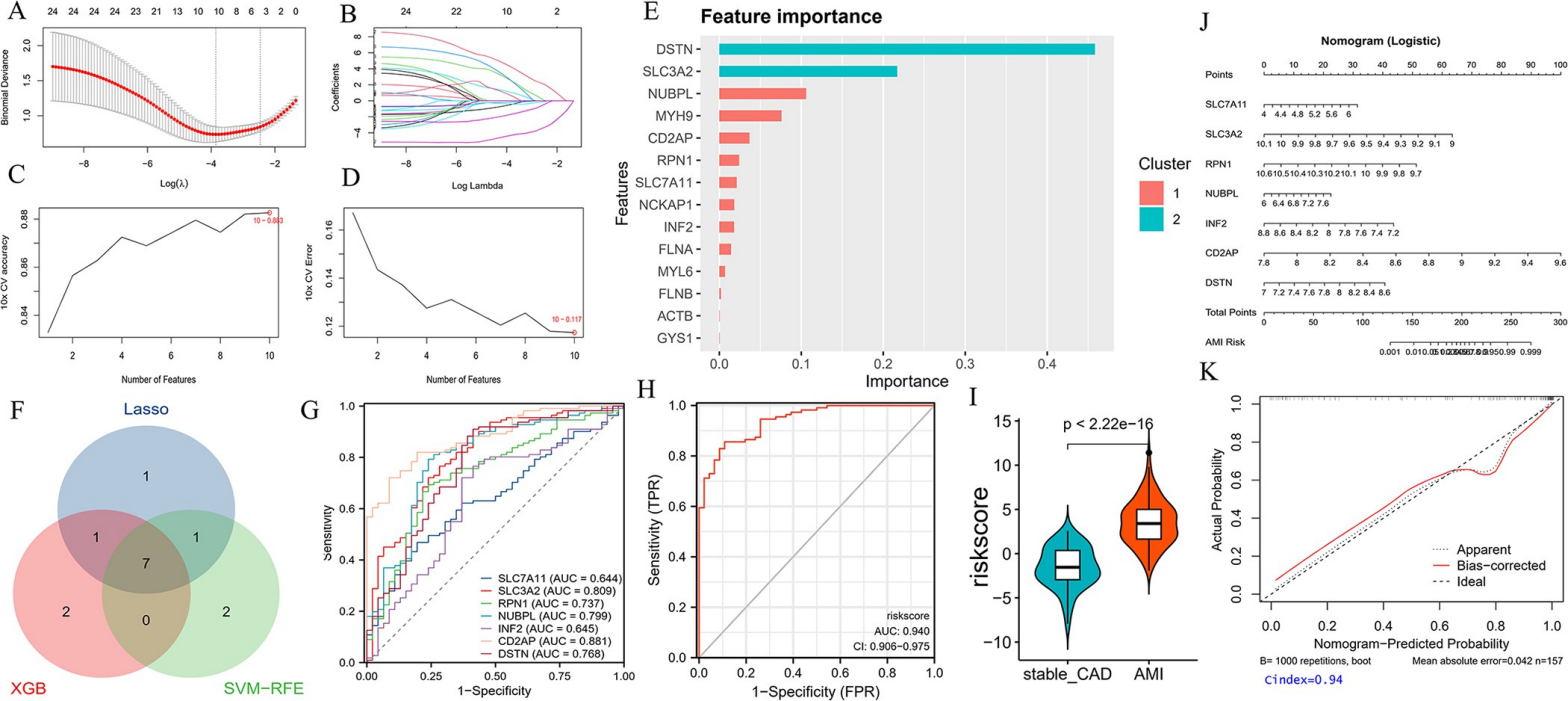
DE-DSRGs were identified as diagnostic genes for AMI. A, B. The LASSO algorithm with 10-fold cross-validation for penalty parameter tuning was used to filter out 10 AMI-related features. C, D. The SVM-RFE algorithm screens the DSRG to determine the optimal combination of feature genes. E. The XGB algorithm screens DSRGs to determine the importance of signature genes. F. A Venn diagram of three machine learning screens for feature genes. G. ROC curve of hub genes. H. ROC curve of risk score. I. The violin diagram shows the risk score of AMI and stable_CAD. J. Nomogram for predicting the risk score of hub genes. K. Calibration curve for logistic regression.

To investigate the diagnostic value of DSRGs in AMI, we screened and constructed a diagnostic model by machine learning. We filtered to 10 signature genes by the LASSO algorithm when lambda. min was 0.02116 (Fig 2A and 2B). Next, The SVM-RFE was employed to rank the importance of DSRGs, selecting the top 10 signature genes with an AUC of 0.883 (Fig 2C, 2D and S4 Table). Fig 2E showed the DSRGs feature importance ranking by the XGBoost algorithm (S5 Table). We obtained seven signature genes as hub genes (SLC7A11, SLC3A2, RPN1, NUBPL, INF2, CD2AP, DSTN), overlapping genes screened by three machine learning algorithms (Fig 2F).

Afterwards, the ischemic events prediction efficacy of hub genes was assessed through ROC curves in GSE59867. The findings revealed that SLC3A2 (AUC = 0.809), NUBPL (AUC = 0.799), CD2AP (AUC = 0.881), and DSTN (AUC = 0.768) possessed promising diagnostic potential. In addition, we utilized multifactor logistic regression to construct a risk score model based on hub gene expression. The final risk score = -47.0693–1.3886*SLC7A11+5.6012*SLC3A2+5.5376*RPN1-1.2138*NUBPL+2.6503*INF2-5.3961*CD2AP-2.4710*DSTN. The ROC curves indicated that the risk score had a strong diagnostic potential (Fig 2H). Additionally, the risk score for AMI is higher compared to stable CAD (Fig 2I). A line diagram model based on hub genes was constructed (Fig 2J). Furthermore, the calibration curves demonstrated that the line diagram model exhibited precise predictive capability (Fig 2K).

### 3.4 Validation of hub genes and diagnostic models

**Fig 3:**
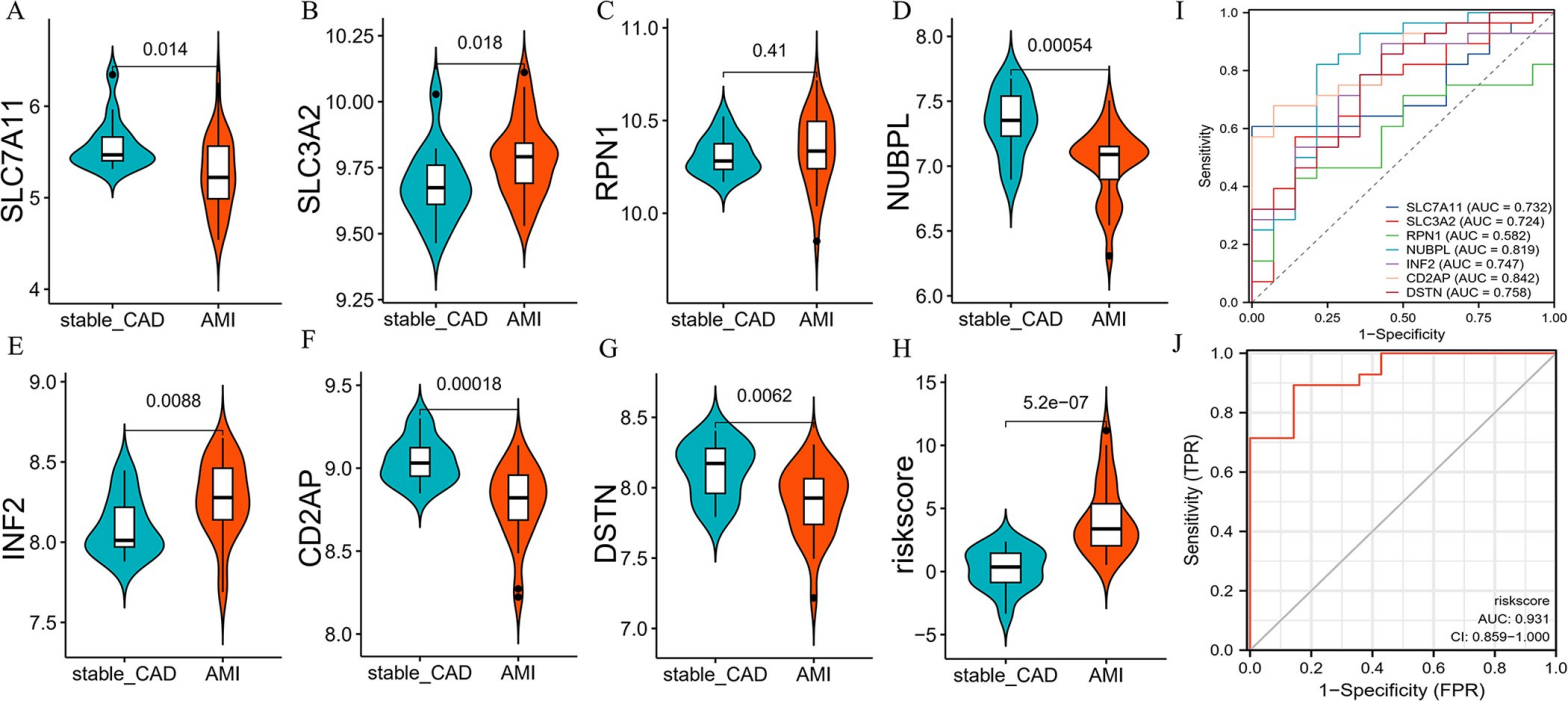
Validation of hub genes and diagnostic models in GSE62646. A-H. The expression pattern of hub genes in GSE62646. I, J. ROC curve of hub genes and risk score in GSE62646.

We also validated hub genes and risk models in the validation set. The expression patterns and diagnostic value of hub genes were consistent with those in the training set (Fig 3A–3G and 3I). In addition, there was a higher risk score in the AMI (Fig 3H), which was also consistent with the training set results, and the risk score AUC was 0.931 (Fig 3J).

### 3.5 Prognostic value of hub genes

**Fig 4:**
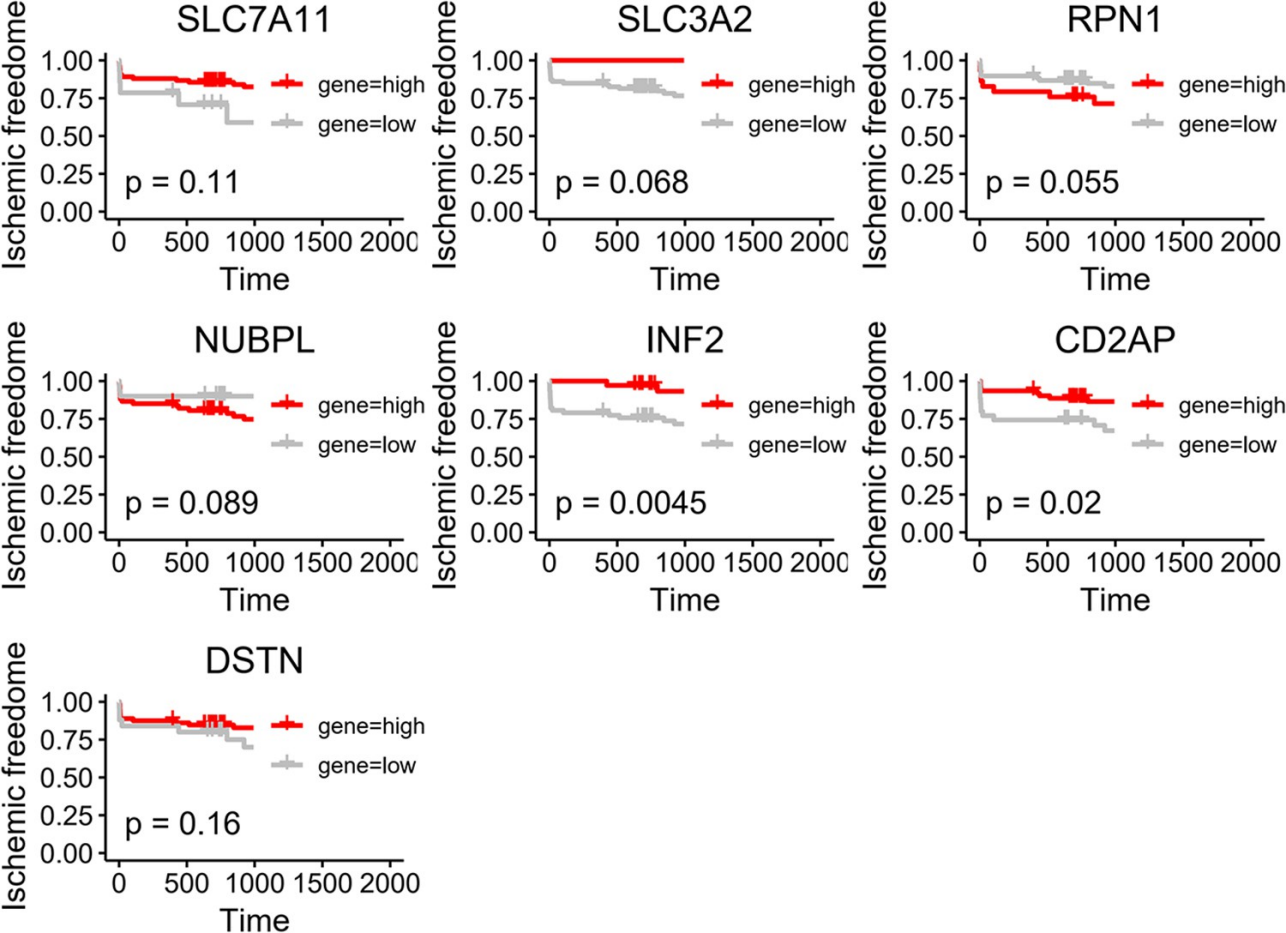
K–M curve of hub genes.

The K-M algorithm was employed to evaluate the prognostic value of hub genes in GSE21545. The results suggested that the high-expression group of INF2 and CD2AP had a better prognosis (Fig 4).

### 3.6 Immune micro environment analysis

**Fig 5:**
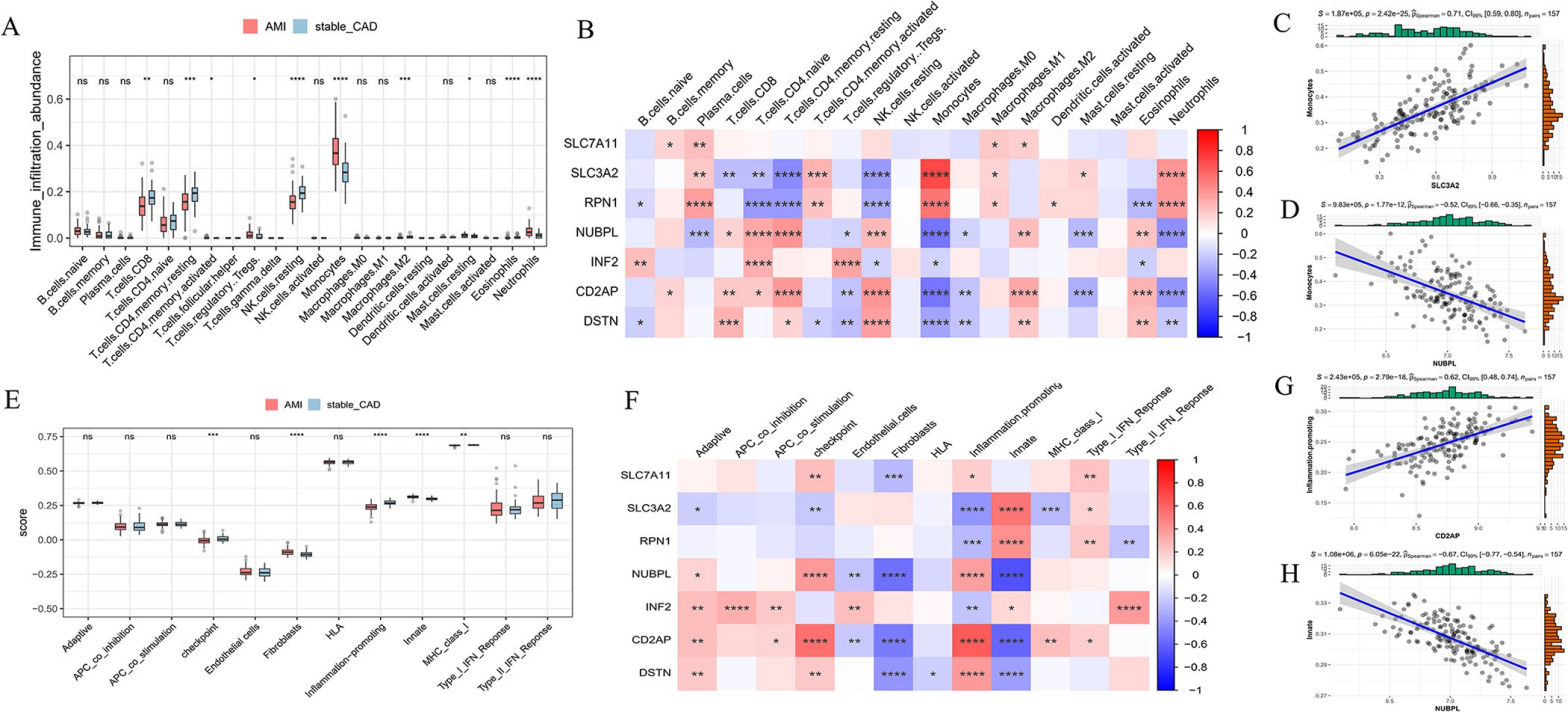
Immune landscape analysis. A. Immune infiltration pattern between AMI and stable_CAD. B. Heatmap of hub genes and immune cell correlation. C. Scatter plot of the correlation between SLC3A2 and Monocytes. D. Scatter plot of the correlation between NUBPL and Monocytes. E. Immune function scores between AMI and stable_CAD. F. Heatmap of hub genes and immune function scores. G. Scatter plot of the correlation between CD2AP and Inflammation promoting. H. Scatter plot of the correlation between NUBPL and Innate.

Studies have shown that immune regulation plays an important role in the process of AMI. Then, we explored the relationship between hub genes and the immune microenvironment. The CIBERSORT algorithm was used to calculate sample immune infiltration abundance. T cells regulatory Tregs, monocytes, neutrophils, and macrophages M2 had higher abundances of immune cell infiltrates in AMI compared with controls; In contrast, T cells CD8, T cells CD4 memory resting, NK cells resting have a lower abundance of immune cell infiltration (Fig 5A). Correlation analysis has revealed a robust positive correlation between SLC3A2 and monocytes, while indicating a strong negative correlation between NUBPL and monocytes (Fig 5B–5D).

In addition, we analyzed the relationship between hub genes and immune function. Compared to the control group, Fibroblasts and Innate scores were higher in AMI, while Checkpoint, Inflammation-promoting, and MHC_class_I scores were lower in AMI (Fig 5A). Correlation analysis showed a strong positive correlation between CD2AP and inflammation promoting, and a strong negative correlation between NUBPL and innate (Fig 5F–5H).

### 3.7 Construction of ceRNA, small molecule drug networks, and TFs with hub genes

**Fig 6:**
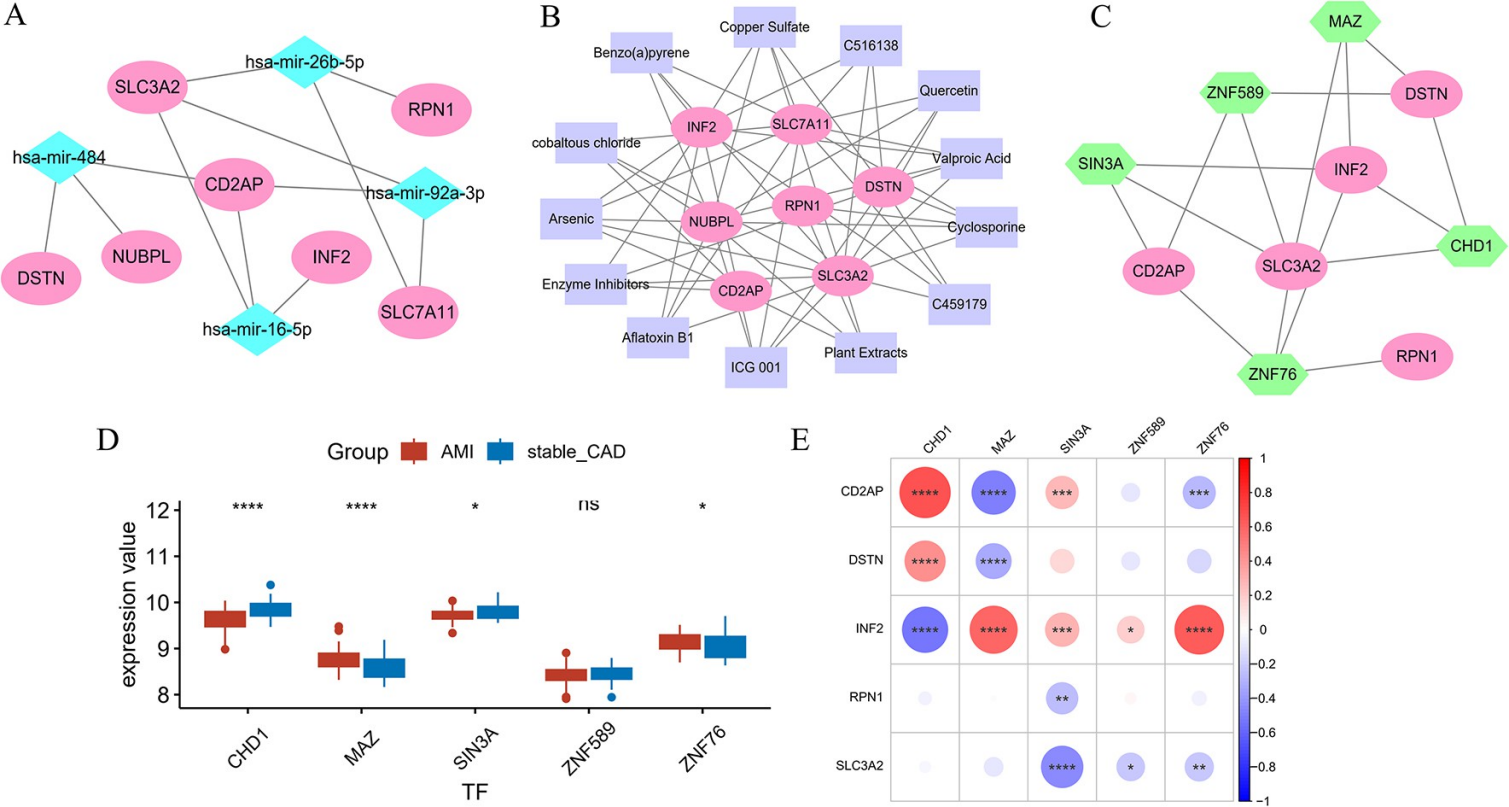
A. Diagram of the interaction network between hub genes and miRNAs. B. Diagram of the interaction network between hub genes and small molecule drugs. C. Diagram of the interaction network between hub genes and TFs. D. Box plots of the expression patterns of TFs in AMI. E. Heatmap of hub genes and TFs.

In order to create networks that incorporate ceRNA, small molecule drugs, and TFs, the NetworkAnalyst database was employed. We excavated 249 miRNAs, 242 compounds, and 161 TFs, interacting with hub genes (S6–S8 Tables). These miRNAs with a degree greater than or equal to 3 were hsa-mir-484, hsa-mir-16-5p, hsa-mir-26b-5p, and hsa-mir-92a-3p (Fig 6A). These TFs with a degree greater than or equal to 3 were SIN3A, ZNF589, MAZ, ZNF76, and CHD1 (Fig 6C). And there are 13 compounds with a degree of 4 or greater (Fig 6B). Then, we studied the expression of TFs in AMI and the correlation between TFs and hub genes. MAZ and ZNF76 were highly expressed, whereas CHD1 and SIN3A were low expressed in AMI (Fig 6D). Furthermore, correlation analysis demonstrated a significant positive correlation between CHD1 and CD2AP, as well as between MAZ and ZNF76 with INF2. (Fig 6E).

### 3.8 Validation in a single-cell dataset

**Fig 7:**
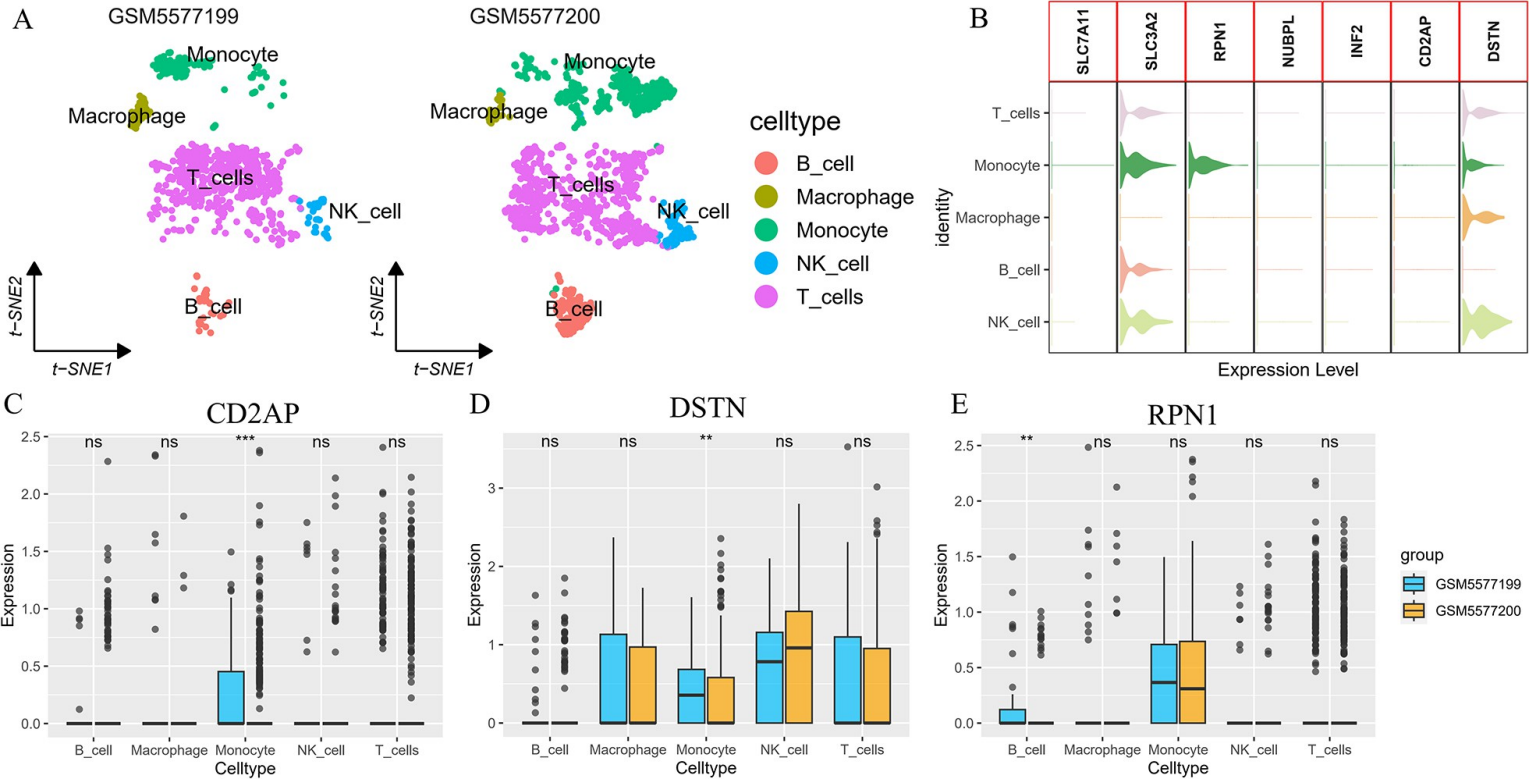
Hub genes expression in single-cell data. A. The tSNE plots of the five cell clusters in GSE184073. B. The stacked violin plot of hub genes expression in GSE184073. C-E. Differential expression of CD2AP, DSTN, and RPN1 between AMI and stable CAD in 5 cell clusters.

After QC filtering, the GSE184073 dataset yielded 23,320 genes and 2,223 cells. We obtained five major cells via SingleR autoannotation, including B cells, macrophages, monocytes, NK cells, and T cells (Fig 7A). Subsequently, the expression of hub genes was analyzed in different cells. SLC3A2 was highly expressed in B cells, monocytes, NK cells, and T cells, DSTN was highly expressed in macrophages, monocytes, NK cells, and T cells, and RPN1 was only highly expressed in monocytes (Fig 7B). Compared with the acute coronary syndrome (ACS), CD2AP and DSTN were higher expressed in monocytes cell of stable angina pectoris (SAP), and RPN1 was higher expressed in B cell of SAP (Fig 7C–7E).

### 3.9 Regulation mode of hub genes

**Fig 8:**
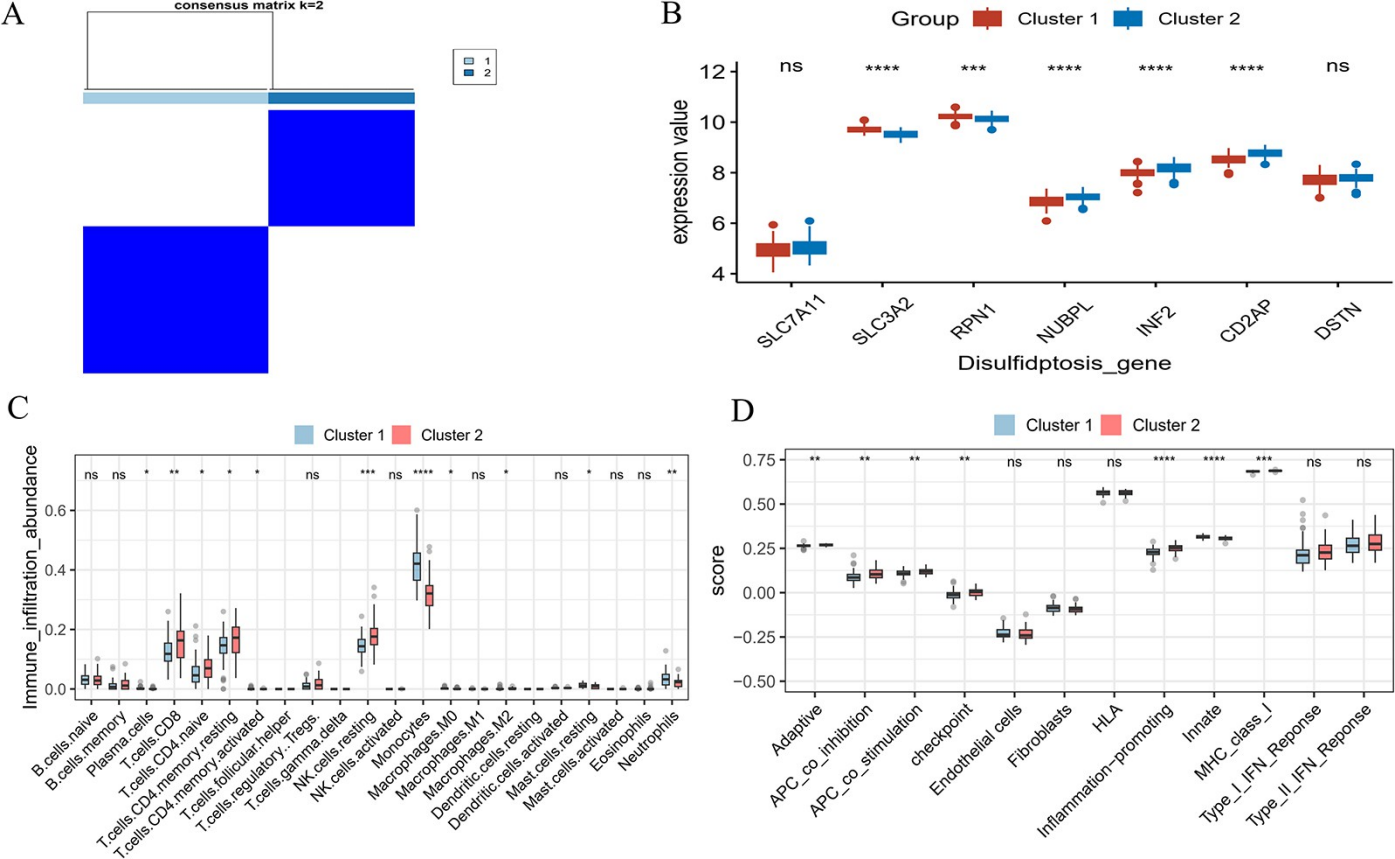
Subtype classification of AMI patients based on hub genes. A. Unsupervised consensus clustering matrix and optimal clustering. B. Differential expression of hub genes between Cluster 1 and Cluster 2. C. Immune infiltration pattern between Cluster 1 and Cluster 2. D. Immune function scores between Cluster 1 and Cluster 2.

In order to further explore the regulatory mode of hub genes, we adopted the consistent clustering method to classify the disease subtypes of AMI patients. It can be seen that AMI patients were significantly divided into two groups when K = 2 (Fig 8A). In Cluster 1, SLC3A2 and RPN1 exhibited high expression levels, whereas Cluster 2 showed high expression levels of NUBPL, INF2, and CD2AP. (Fig 8B). Immunoassay suggested that Monocytes and neutrophils were strongly infiltrated in Cluster 1, and T cells CD8, T cells CD4 naive, T cells CD4 memory resting, and NK cells resting in Cluster 2 had a higher degree of immune infiltration (Fig 8C). In terms of immune function, Innate had a higher score in Cluster 1. Conversely, Adaptive, APC_co_inhibition, APC_co_stimulation, checkpoint, Inflammation−promoting, and MHC_class_I had higher scores in Cluster 2 (Fig 8D).

### 3.10 Biological properties between Cluster 1 and Cluster 2

**Fig 9:**
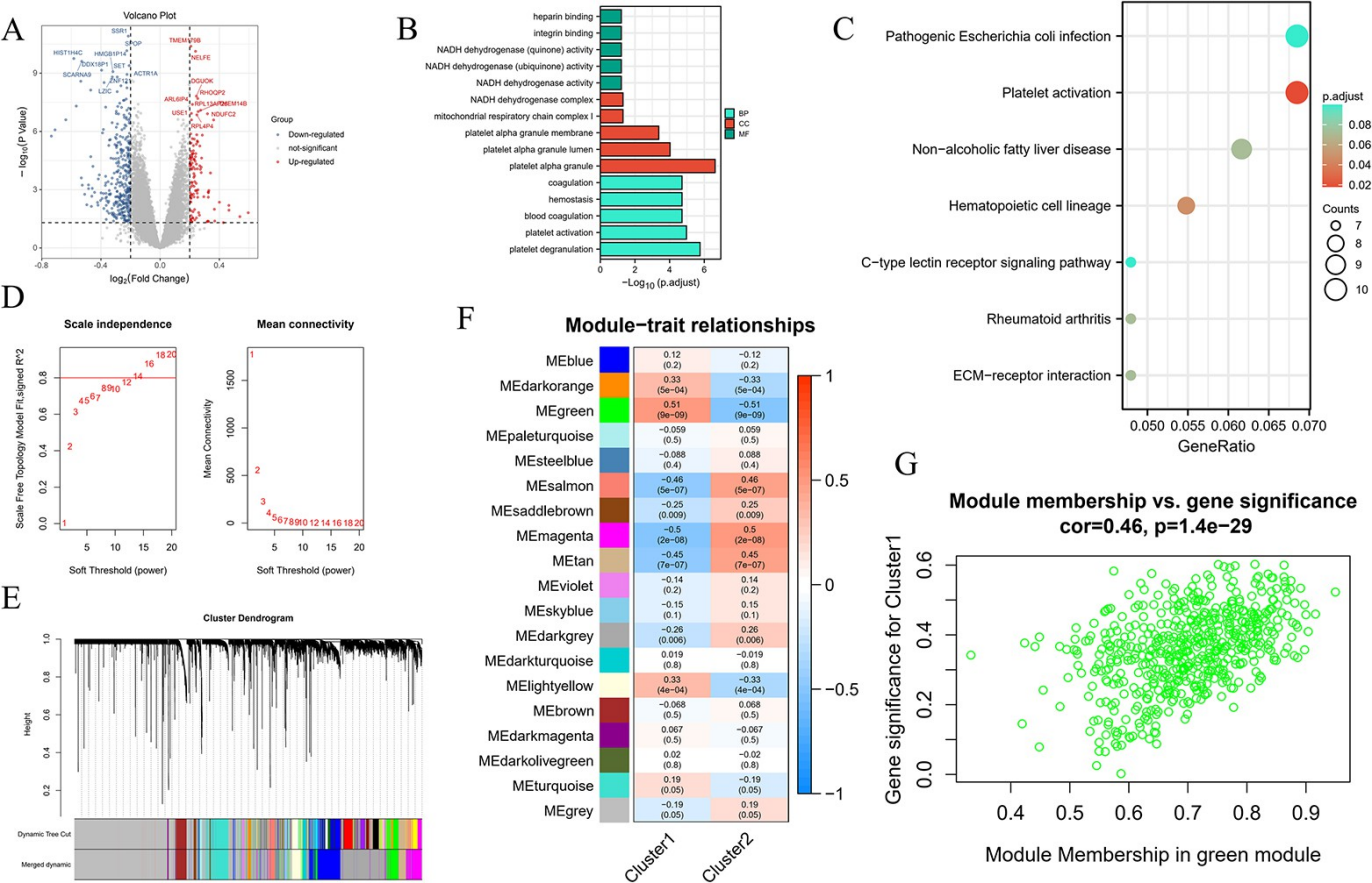
Regulatory model of hub genes. A. Volcano map of differential genes Cluster 1 vs. Cluster 2. B. GO analysis results in the histogram display. C. KEGG enrichment analysis results in bubble chart display. D. Scale-free ft index and average connectivity analysis for different soft threshold powers. E. The average linkage hierarchical clustering method was used to construct a gene dendrogram. F. Heatmap of the correlation between different modules and disease subtypes. G. Scatter plot of the correlation between the green module and Cluster 1.

Differential analysis showed that 445 differential genes were identified as Cluster 1 vs. Cluster 2 (Fig 9A). Functional enrichment analysis revealed that coagulation, hemostasis, blood coagulation, platelet activation, and platelet degranulation were significantly enriched in BP (Fig 9B); NADH dehydrogenase complex, mitochondrial respiratory chain complex I, platelet alpha membrane, NADH dehydrogenase complex, mitochondrial respiratory chain complex I, platelet alpha membrane, platelet alpha granule lumen, and platelet alpha granule was significantly enriched in CC (Fig 9B); heparin-binding, integrin binding, NADH dehydrogenase (quinone) activity, NADH dehydrogenase (ubiquinone) activity, and NADH dehydrogenase activity was significantly enriched in MF (Fig 9B); pathogenic Escherichia coli infection, Platelet activation, Non−alcoholic fatty liver disease, Hematopoietic cell lineage, and C−type lectin receptor signaling pathway were significantly enriched in the KEGG pathway (Fig 9C).

The WGCNA method was used to identify the gene-gene modules associated with the two models. When the soft threshold was 14, nineteen modules were obtained after clustering and merging (Fig 9D and 9E). Cluster 1 had the highest positive correlation with the green module, and Cluster 2 had the highest positive correlation with the magenta module (Fig 9F and 9G).

### 3.11 Validation of Hub genes in vitro

**Fig 10:**
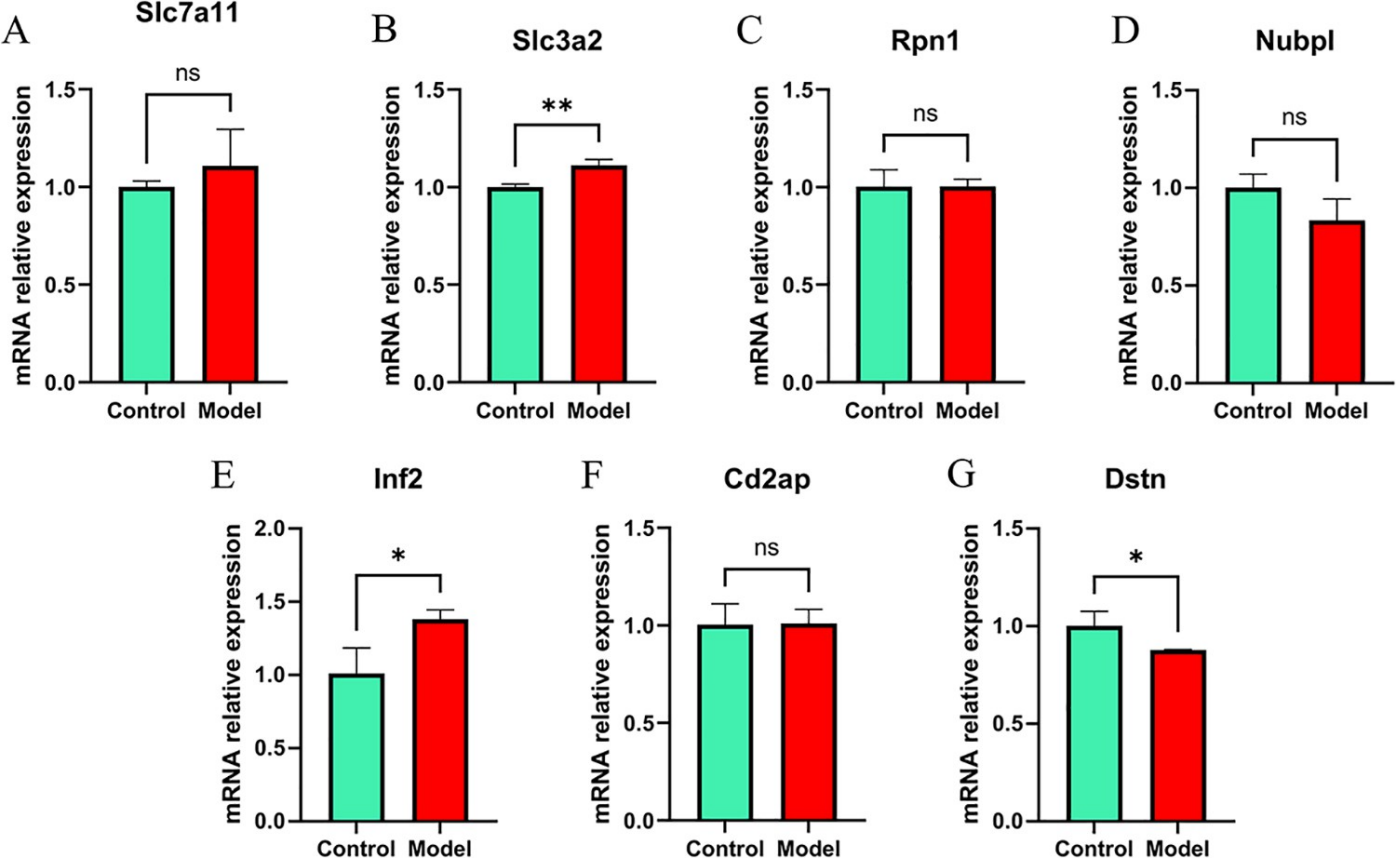
Validation of Hub genes by qPCR. mRNA relative expession of A.Slc7a11, B.Slc3a2, C.Rpn1, D.Nubpl, E.Inf2, F.Cd2ap, G.Dstn.

To further verify the results of bioinformatics analysis, we verified the expression of hub genes in vitro by qPCR. Conspicuously, Slc3a2 and Inf2 were significantly up-regulated and Dstn was significantly down-regulated in the hypoxic model (Fig 10).

## 4. Discussion

Disulfidptosis is a newly discovered method of cell death [8]. Currently, little is known about its role in disease initiation and progression. This study utilized bioinformatics methods to investigate the diagnostic and prognostic significance of DSRGs in AMI, and shed light on their potential regulatory mechanisms in this condition. It is the first study to explore these aspects of DSRGs in AMI.

DSRGs were obtained from previous literatures [8], and the results of go and KEGG analysis showed that they mainly participated in cytoskeleton formation, cell adhesion, platelet condensation, platelet activation. In the early stage of atherosclerosis (AS), the recruitment and adhesion of monocytes can induce the inflammatory response of vascular plaques and promote the development of AS [20]. Adhesion of inflammatory cells after MI occurs increases the risk of heart reinfarction [21]. This indicates that the attachment of inflammatory cells is a crucial factor in the etiology and prognosis of AMI [22]. In addition, platelets play a critical role in the occurrence and development of AMI, which has been widely recognized by scholars [23–25]. Under physiological conditions, platelets do not adhere to the intact endothelial monolayers, and when AMI occurs, atherosclerotic plaques rupture, inducing platelet aggregation and activation, and initiating platelet-mediated thrombosis [23, 26, 27].

Machine learning algorithms identified seven DSRGs (SLC7A11, SLC3A2, RPN1, NUBPL, INF2, CD2AP, DSTN) with significant diagnostic value, which were further utilized in constructing a diagnostic model using logistic regression. The model demonstrated a strong diagnostic potential (AUC = 0.940). The validation results were largely consistent with the findings of previous bioinformatics analyses. This suggests that our diagnostic model has some potential value for clinical application. In addition, INF2 and CD2AP had prognostic value for the occurrence of ischemic events.

SLC7A11, a cystine/glutamate reverse transporter, is involved in the uptake of cystine for glutathione biosynthesis and antioxidant defense, and is involved in the development of a variety of diseases by inhibiting ferroptosis [28–30]. There is evidence that SLC7A11 plays a protective role in myocardial ischemia/reperfusion, and the mechanism is that overexpressed SCL7A11 inhibits ferroptosis in cardiomyocytes [30, 31]. SLC3A2 is an endoplasmic reticulum stress regulator. Studies have shown that SLC3A2 may induce endoplasmic reticulum stress by activating ATF4, ATF6 and XBP1 [32], and endoplasmic reticulum stress can induce cardiomyocyte apoptosis in AMI [33, 34]. RPN1 is involved in the regulation of protein ubiquitination [35]. Studies have shown that deubiquitination regulates myocardial fibrosis after myocardial ischemia-reperfusion injury [36]. Mitochondrial dysfunction during acute ischaemia and reperfusion injury is a key determinant of cell death after AMI. Therefore, mitochondrial quality control and mitochondrial function are hot topics in myocardial ischemia/reperfusion therapy. INF2 is involved in mitochondrial division and NUBPL is involved in the assembly of mitochondrial respiratory chain complex I, suggesting that both play important roles in mitochondrial quality control and function [37].

The results of immunolandscape analysis revealed that monocytes infiltration degree was higher in AMI. Single cell sequencing analysis showed that CD2AP and DSTN were significantly different in monocytes. Research reports that after AMI, mice heart number of monocytes and macrophages in infarction area expansion, phenotypic change especially. In vivo microscopy showed that monocytes began to recruit to the infarct site 30 minutes after myocardial infarction, and this initial recruitment was quickly replaced by neutrophil infiltration [38]. Recent evidence suggests that these sentinel infiltrating monocytes may play a role in neutrophil recruitment [39]. The recruitment of monocytes peaked at 3–5 days after AMI, during which monocytes mainly exhibited inflammatory cytokines, such as TNFα, which played a pro-inflammatory role [40]. The inflammatory response at the beginning of an AMI is designed to remove necrotic cell debris from the infarct area [40]. However, excessive and persistent inflammatory responses lead to enlargement of the AMI infarct area and poor remodeling in later periods [41]. Clinical trials have shown that nonspecific anti-inflammatory therapy (such as glucocorticoids and nonsteroidal anti-inflammatory drugs) in the early stage of AMI can reduce infarct areas [42, 43]. These results indicate that monocytes play an essential role in the early stage of AMI, and targeted monocyte recruitment pathway may be an effective therapeutic pathway for AMI. Consistent cluster analysis based on hub genes can divide AMI patients into two clinical subtypes. The presence of monocyte infiltration in Cluster 1 was significantly higher than that in Cluster 2. Considering that excessive inflammatory response is not conducive to AMI patients, we speculated that the prognosis of Cluster 2 might be better than Cluster 1.

Furthermore, we have constructed the ceRNA network of hub genes and selected four miRNAs that interact closely with hub genes. Literature analysis showed that hsa-miR-16-5p was highly expressed in AMI, which may be involved in regulating the repair of endothelial injury [44]. However, miR-26b-5p is associated with poor left ventricular remodeling after myocardial infarction, but the specific mechanism remains unclear [45]. Interestingly, there are few studies on mir-484 and miR-92a-3p in AMI.

To explore the mechanism of disruption of hub gene expression, we analyzed the TFs of hub genes and their expressions. The results indicate a positive correlation between DSTN and CHD1 expression, and a positive correlation between INF2 and MAZ/ZNF76 expression. As such, we speculated that the expression disorders of DSTN and INF2 might be caused by the expression disorders of TFs. Other changes in hub genes are more likely to involve regulatory mechanisms other than TFs, such as DNA methylation or enhancers.

## 5. Conclusion

In summary, the research provided a comprehensive analysis of the molecular alterations and gene interactions in disulfidptosis in AMI. In addition, we also constructed a diagnostic prediction model for AMI and classified the clinical subtypes of AMI patients based on seven genes, providing a new perspective for the prevention and treatment of clinical AMI. Of course, this study also has many shortcomings, including the absence of clinical sample or animal-level validation, as well as being limited by the sample size of public databases. Therefore, prospective clinical studies with larger samples will be needed.

## Supporting information

S1 Table The correlation coefficient between DSRGs. (XLSX)

S2 Table The significance of correlation between DSRGs. (XLSX)

S3 Table GO and KEGG clustering analysis results of DSRGs. (XLSX)

S4 Table Results of SVM-RFE algorithm in screening feature genes. (XLSX)

S5 Table Results of XGBoost algorithm in screening feature genes. (XLSX)

S6 Table The ceRNA network of hub genes. (XLSX)

S7 Table Network of interactions between hub genes and small molecule compounds. (XLSX)

S8 Table Network of interactions between hub genes and TFs. (XLSX)

## Abbreviations

DSRGs: disulfidptosis related genes
AMI: acute myocardial infarction
stable_CAD: stable coronary artery disease
GEO: gene expression omnibus
PPI: protein–protein interaction
XGBoost: extreme gradient boosting
LASSO: least absolute shrinkage and selection operator
SVM-RFE: support vector machine—recursive feature elimination
AUC: area under curve
ROC: receiver operating characteristic
K-M: Kaplan–Meier
GO: gene ontology
KEGG: kyoto encyclopedia of genes and genomes
CIBERSORT: cell-type identification by estimating relative subsets of RNA transcript
ssGSEA: single-sample gene set enrichment analysis
ceRNA: competing endogenous RNA
TFs: transcription factors
WGCNA: weighted correlation network analysis
TOM: topology overlap matrix
QC: quality control
PCA: principal component analysis
tSNE: t-distributed stochastic neighbor embedding
BP: biological process
CC: cellular component
MF: molecular function
